# From Strain Ratio to Shear-Wave Elastography: Evolving Role of EUS in Liver Fibrosis Assessment

**DOI:** 10.5152/tjg.2026.25663

**Published:** 2026-02-19

**Authors:** Yasemin Kaldirim Armutcuoglu, Dilara Turan Gokce

**Affiliations:** 1 Department of Gastroenterology, Marmara University School of Medicine, İstanbul, Turkiye; 2 Department of Gastroenterology, Ankara Universtiy School of Medicine, Ankara, Türkiye

Dear Editor,

We read with great interest the article by Duman et al. entitled “Usefulness of Endoscopic Ultrasound Strain Elastography for Measuring Liver Stiffness and the Role of Blood Cytokeratin-18 Levels as a Surrogate Marker of Fibrosis.”^1^ The study provides valuable insight into the application of endoscopic ultrasound (EUS)-based elastography for hepatic fibrosis assessment.

Strain elastography (SE) estimates tissue deformation in response to mechanical or physiological compression and displays elasticity on a color-coded map. The strain ratio (SR), a semi-quantitative index comparing the target parenchyma with a reference area, reflects relative stiffness rather than an absolute value.^2^ Although conceptually valuable, SE is highly operator dependent, influenced by variability in reference tissue selection, and lacks standardized acquisition protocols, which limit its reproducibility in diffuse liver disease.^2^^,^^3^ Moreover, because SE measures stiffness indirectly, it demonstrates limited correlation with histologic fibrosis stage and other noninvasive techniques, restricting its clinical utility primarily to the detection of advanced disease rather than full fibrosis staging.^3^

In contrast, shear-wave–based elastography techniques generate acoustic pulses that induce shear waves within the liver, enabling quantitative stiffness measurement in kilopascals (kPa) or meters per second (m/s). These modalities, including transient elastography (TE), point shear-wave elastography (pSWE), and two-dimensional shear-wave elastography (2D-SWE), provide objective and reproducible measurements without the need for reference tissue selection. They demonstrate stronger agreement with histologic staging and with established noninvasive techniques such as magnetic resonance elastography (MRE).^2^^,^^4^ The 2024 World Federation for Ultrasound in Medicine and Biology Guidelines emphasize that both pSWE and 2D-SWE offer standardized quantitative liver stiffness assessment and that strain-based methods are no longer recommended for fibrosis staging.^3^ Although shear-wave techniques require dedicated equipment and operator training, their integration into EUS platforms has expanded diagnostic capabilities, particularly in patients with limited transabdominal evaluation.^4^^-^^7^ Nonetheless, reliable EUS-guided shear-wave elastography (EUS-SWE) acquisition requires specific technical expertise, including stable echoendoscope positioning, consistent region-of-interest placement within the left hepatic lobe, and careful minimization of motion artifacts, factors that contribute to a meaningful learning curve and may affect early implementation in centers without established experience.^3^^,^^5^^,^^7^

In the study by Duman et al, participants were evaluated using EUS strain elastography. The authors reported significantly higher SR values in patients with cirrhosis compared with those with non-cirrhotic chronic liver disease (CLD) and healthy controls. Strain ratio thresholds of 5.67 and 10.65 showed high sensitivity and specificity in differentiating cirrhosis from non-cirrhotic CLD and from healthy individuals, respectively.^1^ These findings confirm that EUS-based SE can distinguish advanced fibrosis; however, recent evidence indicates that EUS-SWE provides more accurate and standardized quantification.

Diehl et al^5^ demonstrated strong correlations between EUS-SWE, histologic fibrosis stage, and vibration-controlled transient elastography (VCTE) measurements. Similarly, del Valle et al^6^ reported excellent agreement between EUS-SWE and VCTE in cirrhotic cohorts. Wang et al^7^ further showed that EUS-SWE achieves a high technical success rate in patients with obesity or metabolic dysfunction–associated steatotic liver disease, a setting in which transabdominal TE frequently fails. Beyond ultrasound, MRE remains the most accurate noninvasive reference method, producing whole-liver stiffness maps with superior reproducibility and minimal operator dependence.^4^

In conclusion, the study by Duman et al represents an important early contribution to EUS-based hepatic elastography and highlights the growing integration of endoscopic and hepatologic disciplines within the evolving field of endohepatology. As the field advances toward quantitative shear-wave and multiparametric imaging modalities, future multicenter studies integrating EUS-SWE with histology-validated quantitative parameters are warranted to refine and standardize noninvasive fibrosis assessment.

## Data Availability

The data that support the findings of this study are publicly available.
